# Supplementary material for: Analysis of SARS-CoV-2 vertical transmission during pregnancy
Source: Nat Commun. 2020 Oct 12;11:5128. doi: 10.1038/s41467-020-18933-4 (PMC7552412; doi:10.1038/s41467-020-18933-4)
Supplement: Supplementary file 1 — Supplementary Information [file 41467_2020_18933_MOESM1_ESM.pdf]

Analysis of SARS-CoV-2 vertical transmission during pregnancy

Claudio Fenizia[ǂ], Mara Biasin [ǂ] *et al.*

Supplementary information

Supplementary Table I. SARS-CoV-2 Real-time PCR Panel Primers and Probes

**Name**	**Description**	**Oligonucleotide Sequence (5'>3')**	**Label**
2019-nCoV_N1-F	2019-nCoV_N1 Forward Primer	5'-GAC CCC AAA ATC AGC GAA AT-3'	None
2019-nCoV_N1-R	2019-nCoV_N1 Reverse Primer	5'-TCT GGT TAC TGC CAG TTG AAT CTG-3'	None
2019-nCoV_N1-P	2019-nCoV_N1 Probe	5'-FAM-ACC CCG CAT TAC GTT TGG TGG ACC-BHQ1-3'	FAM, BHQ-1
2019-nCoV_N2-F	2019-nCoV_N2 Forward Primer	5'-TTA CAA ACA TTG GCC GCA AA-3'	None
2019-nCoV_N2-R	2019-nCoV_N2 Reverse Primer	5'-GCG CGA CAT TCC GAA GAA-3'	None
2019-nCoV_N2-P	2019-nCoV_N2 Probe	5'-FAM-ACA ATT TGC CCC CAG CGC TTC AG-BHQ1-3'	FAM, BHQ-1
RP-F	RNAse P Forward Primer	5'-AGA TTT GGA CCT GCG AGC G-3'	None
RP-R	RNAse P Reverse Primer	5'-GAG CGG CTG TCT CCA CAA GT-3'	None
RP-P	RNAse P Probe	5'-FAM – TTC TGA CCT GAA GGC TCT GCG CG – BHQ-1-3'	FAM, BHQ-1

TaqMan® probes are labeled at the 5'-end with the reporter molecule 6-carboxyfluorescein (FAM) and with the quencher, Black Hole Quencher 1 (BHQ-1) (Biosearch Technologies, Inc., Novato, CA) at the 3'-end.
