# Supplementary material for: Analysis of SARS-CoV-2 vertical transmission during pregnancy
Source: Nat Commun. 2020 Oct 12;11:5128. doi: 10.1038/s41467-020-18933-4 (PMC7552412; doi:10.1038/s41467-020-18933-4)
Supplement: Supplementary file 3 — Reporting Summary [file 41467_2020_18933_MOESM3_ESM.pdf]

nature research

Corresponding author(s): Valeria Savasi, NCOMMS-20-28958

Last updated by author(s): Aug 31, 2020

Reporting Summary

Nature Research wishes to improve the reproducibility of the work that we publish. This form provides structure for consistency and transparency in reporting. For further information on Nature Research policies, see our Editorial Policies and the Editorial Policy Checklist.

Statistics

For all statistical analyses, confirm that the following items are present in the figure legend, table legend, main text, or Methods section.

n/a	Confirmed	
☐	☒	The exact sample size (n) for each experimental group/condition, given as a discrete number and unit of measurement
☐	☒	A statement on whether measurements were taken from distinct samples or whether the same sample was measured repeatedly
☐	☒	The statistical test(s) used AND whether they are one- or two-sided<br>*Only common tests should be described solely by name; describe more complex techniques in the Methods section.*
☒	☐	A description of all covariates tested
☒	☐	A description of any assumptions or corrections, such as tests of normality and adjustment for multiple comparisons
☒	☐	A full description of the statistical parameters including central tendency (e.g. means) or other basic estimates (e.g. regression coefficient) AND variation (e.g. standard deviation) or associated estimates of uncertainty (e.g. confidence intervals)
☐	☒	For null hypothesis testing, the test statistic (e.g. F, t, r) with confidence intervals, effect sizes, degrees of freedom and P value noted<br>*Give P values as exact values whenever suitable.*
☒	☐	For Bayesian analysis, information on the choice of priors and Markov chain Monte Carlo settings
☒	☐	For hierarchical and complex designs, identification of the appropriate level for tests and full reporting of outcomes
☒	☐	Estimates of effect sizes (e.g. Cohen's d, Pearson's r), indicating how they were calculated

Our web collection on statistics for biologists contains articles on many of the points above.

Software and code

Policy information about availability of computer code

Data collection	Galileo software data was used to collect the clinical data
Data analysis	For the array analyses the Gene Globe Data Analysis by Qiagen was used. For statistic analyses, SPSS Statistics Version 26.0 and GraphPad Prism 8 were used.

For manuscripts utilizing custom algorithms or software that are central to the research but not yet described in published literature, software must be made available to editors and reviewers. We strongly encourage code deposition in a community repository (e.g. GitHub). See the Nature Research guidelines for submitting code & software for further information.

Data

Policy information about availability of data

All manuscripts must include a data availability statement. This statement should provide the following information, where applicable:
- Accession codes, unique identifiers, or web links for publicly available datasets
- A list of figures that have associated raw data
- A description of any restrictions on data availability

All data that support the findings of this study are available from the corresponding author upon reasonable request.

Field-specific reporting

Life sciences study design

All studies must disclose on these points even when the disclosure is negative.

Sample size	No sample size calculation was performed. During the pandemic emergency spanning between March and April 2020 all the pregnant SARS-CoV-2-infected women seeking for care at the hospitals mentioned in the manuscript were enrolled in the study. However, the data presented refer only to those whose we were able to garatee a proper tissue collection and storage. Moreover, for reference in immunological analyses a SARS-CoV-2 negative woman was inclede in the study.
Data exclusions	No data were excluded from the analyses
Replication	Because of importance of the data, all the SARS-CoV-2 detection analyses were performed in duplicate in two independent runs. Such qualitative technical replicates was meant to be a confirmation of previously obtained results.
Randomization	Randomization was not relevant for the study. The only variable taken into consideration was the positivity to SARS-CoV-2 nasopharyngeal swab . Therefore, no allocatio was performed
Blinding	All biological samples were anonymized due to ethical purposes, therefore, they were analysed blinded. Investigators who collected the biological specimens were not blinded. As SARS-CoV-2 positive patients were contained in dedicated hospitals, blinding was not possible.

Reporting for specific materials, systems and methods

We require information from authors about some types of materials, experimental systems and methods used in many studies. Here, indicate whether each material, system or method listed is relevant to your study. If you are not sure if a list item applies to your research, read the appropriate section before selecting a response.

Materials & experimental systems

n/a	Involved in the study	
☐	☒	Antibodies
☒	☐	Eukaryotic cell lines
☒	☐	Palaeontology and archaeology
☒	☐	Animals and other organisms
☐	☒	Human research participants
☒	☐	Clinical data
☒	☐	Dual use research of concern

Methods

n/a	Involved in the study	
☒	☐	ChIP-seq
☒	☐	Flow cytometry
☒	☐	MRI-based neuroimaging

Antibodies

Antibodies used	Bio-Plex Pro™ Human Cytokine 27-plex Assay #M500KCAF0Y
Validation	https://www.bio-rad.com/it-it/sku/m500kcaf0y-bio-plex-pro-human-cytokine-27-plex-assay?ID=m500kcaf0y

Human research participants

Policy information about studies involving human research participants

Population characteristics	All the relevant characteristics are reported in table 1 in the manuscript and include maternal age, BMI, smoking habit, ethnicity, comorbidities, parity, flu vaccination.
Recruitment	During the pandemic emergency spanning between March and April 2020 all the pregnant SARS-CoV-2-infected women seeking for care at the COVID-19 maternity hospitals 'L. Sacco' Hospital (Milan), S. Gerardo Hospital/MBBM Foundation (Monza), and San Matteo Hospital (Pavia) were enrolled in the study. Moreover, for reference in immunological analyses a SARS-CoV-2 negative woman enrolled at COVID-19 free Buzzi maternity Hospital was included in the study. As the only enrollment variable was SARS-CoV-2 positivity and the samples were analized blinded, no self-selection or other conscious biases were introduced.
Ethics oversight	The protocol was approved by the local Medical Ethical and Institutional Review Board (Milan, area 1, #154082020). We obtained informed written consent from the mothers to perform the procedure and analysis, according to CARE guidelines and in compliance with the Declaration of Helsinki principles.

Note that full information on the approval of the study protocol must also be provided in the manuscript.
